# UiO66-NH_2_-TiO_2_/NiF photoanode for photocatalytic fuel cell by towards simultaneous treatment of antibiotic wastewater and electricity generation

**DOI:** 10.1038/s41598-023-49019-y

**Published:** 2023-12-09

**Authors:** Abbas Abbasnia, Roshanak Rezaei Kalantary, Mahdi Farzadkia, Mojtaba Yeganeh, Ali Esrafili

**Affiliations:** 1 Department of Environmental Health Engineering, School of Public Health, Iran University of Medical Sciences, Tehran, Iran; 2 Research Center for Environmental Health Technology, Iran University of Medical Sciences, Tehran, Iran

**Keywords:** Environmental sciences, Chemistry

## Abstract

Environmental destruction, water crisis, and clean energy are among the very important challenges worldwide based on sustainable development goals. Photocatalytic fuel cell, a potential candidate for converting chemical energy into electrical energy through a pollution-free method, holds promise in addressing these challenges. In this regard, we investigated the response of a photoanode covered with UiO66-NH_2_-TiO_2_/NiF on a porous nickel foam as an attractive electrochemical response to remove antibiotics from aqueous solution and simultaneously produce electricity using a one-step hydrothermal synthesis. Nickel foam with its fine structure provides a suitable space for the interaction of light, catalyst, and efficient mass transfer of reactive molecules. It appears that it can be used as a competitive electrode in fuel cells. In order to investigate the properties of the photocatalyst, structural analyses including XRD, FESEM, FTIR, and UV–vis DRS were utilized. Additionally, polarization and electrochemical tests such as chronoamperometry and EIS were measured to further examine the electrochemical features of the PFC photoanode system. The obtained results under optimal conditions (SMZ concentration = 20 ppm, pH = 6, irradiation time = 120 min) were as follows: removal efficiency of 91.7%, P_max_ = 16.98 μW/cm^2^, J_sc_ = 96.75 μA/cm^2^, V_oc_ = 644 mV. The light-induced current flow in UiO66-NH_2_-TiO_2_/NiF exhibited prominent and reproducible photocurrent responses, indicating efficient and stable charge separation in TiO_2_/NiF composite materials, which is a promising strategy for pollutant removal and simultaneous electricity generation.

## Introduction

Environmental destruction, the water crisis, and clean energy based on the goals of sustainable development are among the very important challenges that motivate researchers around the world to address these issues^1–3^. This is due to rapid economic growth, urbanization, and industrialization in recent decades. Consequently, substantial quantities of organic waste are being discharged into water bodies. This not only causes serious pollution of aquatic life but also increases energy consumption for treating polluted water^4–6^. Therefore, it is necessary to remove dangerous pollutants and foster the generation of renewable energy sources or transform our energy production into a sustainable and eco-friendly system. The sustainable solution of recovering and reusing municipal wastewater has been upgrading to address the growing need for a clean and sustainable water supply while also protecting the environment^7,8^.

The misuse and excessive use of sulfonamide antibiotics for treating bacterial infections in humans and animals, as well as their use as growth stimulants in animal husbandry and aquaculture, has raised serious concerns about their discharge into the environment^9^. Sulfonamides (SN) have historically been employed as synthetic antibiotics to combat bacterial infections, resulting in the discharge of their primary metabolites into the environment. Given their notable ability to move through the environment, SNs are frequently detected natural ecosystems. Even small concentrations of SNs in the environment can cause genetic mutations in bacteria, leading to the development of bacteria that are resistant to SNs. Additionally, the existence of SN remainder in the environment presents potential hazards to both the human health and environment. SNs hinder the growth and viability of microorganisms and have a tendency to accumulate in plants because of their strong biological activity^10–12^. Therefore, developing a cost-effective approach for removing antibiotics from municipal wastewater is of utmost.

The fuel cell, which is a promising option for converting chemical energy into electrical energy using a clean method, holds the potential to address these challenges^13,14^. Among the various fuel cell technologies, the photocatalytic fuel cell (PFC) has emerged as a research focus due to its integration of photoelectrocatalysis, solar cells, and fuel cells. This technology offers effective wastewater treatment and simultaneous electricity production. The use of chemical energy through fuel cells can offer several advantages: the ability to recover from water and reuse the treated wastewater, (b) energy savings for wastewater treatment using traditional methods such as aeration, and (c) it can automatically itself to be used as an energy source^14–16^.

A typical fuel cell comprises an anode and cathode connected by an external circuit and a chemical fuel. Electricity is produced as electrons move from the anode to the cathode, driven by the feremi level difference. In the context of a PFC, key components involve a light source, cathode(or photocathode), photoanode, and a suitable electrolyte serving as the chemical fuel^17,18^.

The photoanode is regarded as the primary component in the PFC system since it initiates the operation of the cell^15,19^. PFC systems utilizing TiO_2_ have been extensively investigated and proven successful due to their cost-effectiveness, exceptional catalytic activity, and strong chemical stability. Nevertheless, the currently reported systems suffer from significant limitations in terms of their power generation capacity and efficiency in wastewater treatment. These issues arise from the reliance on a TiO_2_ photoanode that solely responds to UV light^20,21^. To address these drawbacks, numerous studies on PFC systems have sought to overcome this limitation by incorporating a narrow band gap semiconductor. This approach holds promise for enhancing overall effectiveness and simultaneously improving energy production^22,23^. Metal organic frameworks (MOFs) with their well-organized structure, high internal surface area, tunable pores, and abundant electrochemical components with high activity have been widely used. In recent years, the production of MOF compounds with semiconductor compounds that are active in visible light has been recognized as an effective and high-efficiency method^24–26^. MOFs as hosts with high surface area and porous structures and abundant distribution of active sites can provide many paths for photon-induced electron transfer and the advantage of charge carrier separation and increase the efficiency of the photocatalytic process. As a result, a large number of MOFs have been used for various purposes and applications such as the destruction of a wide range of organic pollutants, CO_2_ reduction, adsorption and molecular sensing, etc.^27,28^.

Specifically, zirconium MOFs(UiO-66), exemplified by UiO-66, leverage octahedral building coordination with linear ligands, providing heightened tenability without compromising crystallinity. These MOFs exhibit remarkable thermal stability up to 500 °C and excellent water resistance owing to robust coordination bonds formed through Zr(IV) atoms and carboxylate oxygens in strong-acid-strong-base interactions^29^. The foundational UiO-66 motif, utilizing terephthalic acid (TPA) ligands as linkers, showcases unparalleled thermal stability^30^. Despite its numerous merits, UiO-66 requires ultraviolet radiation for activation due to its relatively wide band gap (~ 3.60 eV)^31^. In the catalytic and photocatalytic domain, modified UiO-66 MOFs display potential as molecular photocatalysts for diverse reactions. The incorporation of various functional groups on the organic ligand is a common strategy to adjust the MOFs' band gap for enhanced visible light absorption. In a study that synthesized a series of isostructures, including UiO-66, UiO-66-NH_2_, UiO-66-(SH)_2_, and UiO-66-(OH)_2_ catalysts, band gap values from reported 3.91 to 2.69eV^32^. UiO-66-NH_2_ has received significant interest owing to its exceptional physicochemical stability, visible light absorption and large specific surface area^33,34^.

Therefore, in this study, our objective is to enhance the photocatalytic activity in the visible light region, we will use an easy one-step synthesis and investigate the properties of UiO-66-NH_2_-TiO_2_ as a photoanode doped on nickel foam (NiF). With respect to its continuous mechanical stability, porous structure and relatively high surface area (in contrast to common substrates such as FTO and ITO), nickel foam can be considered as a highly promising conducting platform for designing photoanodes. Additionally, the spectral absorption of nickel foam in the visible range is significantly higher (around 80% compared to 20% for Transparent Conducting Oxides (TCOs)), which is attributed to the fine microstructure topology of the foam that enables absorption within the hollow structure of the foam^35–37^.

We have also used Cu_2_O/CuO/Cu mesh as photocathode; Because it is abundant in nature and it has also been proven that it is stable due to the incompatibility of the Fermi level with the photoanode and is an effective cathode material^22,38,39^. This study investigates the Effectiveness of the PFC system for the removal of the antibiotic sulfamethoxazole (SMZ) from sulfonamide group with a 0.5 M Na_2_SO_4_ electrolyte by examining the effects of antibiotic concentration, pH, irradiation time, and energy production under xenon light irradiation.

## Material and methods

### Materials

Tetrabutyltitanate ((TiOC_4_H_9_), Ethanol (CH_3_CH_2_OH), Zirconium(IV) chloride (ZrCl_4_), 2-aminoterephthalic acid (BDC-NH_2_), sodium hydroxide (NaOH), Sodium sulfate (Na_2_SO_4_), Hydrofluoric acid (HF), p-Benzoquinone (C_4_H_4_O_2_), Hydrochloric acid (HCl), were purchased from Merck Company (Germany) All the chemicals used were of analytical grade and used without any modifications. Deionized water was utilized whenever necessary throughout the experiments.

### Set-up of photoanode

#### Set-up of TiO_2_/NiF photoanode

The nickel foam was cut into 2 × 3 cm dimensions and then cleaned in acetone, ethanol, hydrochloride solution, deionized water (DI) respectively for 20 min. and then dried in vacuum at 60 °C for 1 h. To prepare TiO_2_/NiF, tetrabutyl titanate was used as a source of titanium. First, Ni foam was immersed in a specific volume/volume ratio of ethanol and tetrabutyl titanate for 20 min and then calcined at 450 °C for 2 h. Finally, 20 ml of tetrabutyl titanate, 25 ml of ethanol, 1 ml of hydrofluoric acid were mixed and stirred for 60 min^40^. After that, the Ni foam and mixed precursor were transferred to a 100 mL Teflon-lined stainless steel autoclave, then heated to 160 °C and kept for 12 h. After natural cooling at room temperature, the prepared samples were thoroughly washed with deionized water and ethanol and then dried at 60 °C for 12 h. Finally, the prepared samples were calcined in a furnace at 550 °C for 2 h to obtain TiO_2_/NiF.

#### Set-up of UiO-66-NH_2_-TiO_2_/NiF photoanode

UiO-66-NH_2_-TiO_2_: UiO-66-NH_2_ was synthesized using the same procedure as described in the previous study, with some modifications incorporated^41^. Dissolve ZrCl_4_, 0.2332 gr in 50 ml of DMF, then add acetic acid (6 ml) dropwise to the solution, and finally add 0.1812 gr of BDC-NH_2_ to the desired solution and stir for 1 h. The desired mixed precursor was transferred to a 100 ml Teflon-lined stainless steel autoclave, then heated to 120 °C in an oven for 24 h. After naturally cooling, the sample was subjected to centrifugation and washed multiple times with anhydrous methanol to remove any remaining DMF. The resulting pale yellow solid was then vacuum dried at 100 °C for 12 h.

UiO-66-NH_2_/TiO_2_ (20% w): In order to create a uniform suspension, a specific quantity of tetrabutyl titanate was dispersed in 100 mL of methanol by stirring for 30 min. Subsequently, an adequate amount of the prepared UiO-66-NH_2_ sample (20% weight ratio) was added to the solution while continuously stirring.

UiO-66-NH_2_-TiO_2_/NiF photoanode: The synthesis of UiO-66-NH_2_-TiO_2_/NiF was coated on the nickel foam surface by one-step hydrothermal method. Now the two solutions of MOF and titanium are mixed and stirred for 1 h. After that, the nickel foam and the desired mixed precursor were transferred to a 100 ml Teflon-lined stainless steel autoclave, then heated to 120 °C in an oven for 24 h. After natural cooling at room temperature, the prepared Ni Foams were thoroughly washed with deionized water and then dried at 60 °C for 12 h. Finally, the prepared samples were calcined in a furnace at 450 °C for 4 h to obtain UiO-66-NH_2_-TiO_2_/NiF. It is noteworthy that in order to achieve the appropriate thickness of the catalyst, this process was repeated three times to maintain the amount of the desired catalyst at 10 mg/cm^2^.

### Set-up of Cu_2_O/CuO/Cu photocathode

A copper sheet measuring 2 × 3 cm is treated with purified ethanol and then immersed in an aqueous solution containing 2.5 mg of sodium hydroxide and 0.125 mg of sodium persulfate for 30 min. The Cu foil is then dried in an oven at 90 °C for 24 h and Cu_2_O/CuO/Cu is achieved through a process of calcination at 450 °C for 2 h^38^.

### Characterization methods

The immobilized photocatalyst is recognized in the following manner: the field-emission scanning electron microscopy (FESEM) with FE-SEM ZEISS Sigma 300, energy dispersive X-ray (EDX), Fourier transform infrared (FTIR) spectroscopy with FTIR Thermo Avatar, the X-ray diffraction (XRD) via Philips PW1730, and, UV–vis diffuse reflectance spectra via (UV–vis DRS) by DRS S_4100 SCINCO.

### Degradation test in PFC system

**Figure 1:**
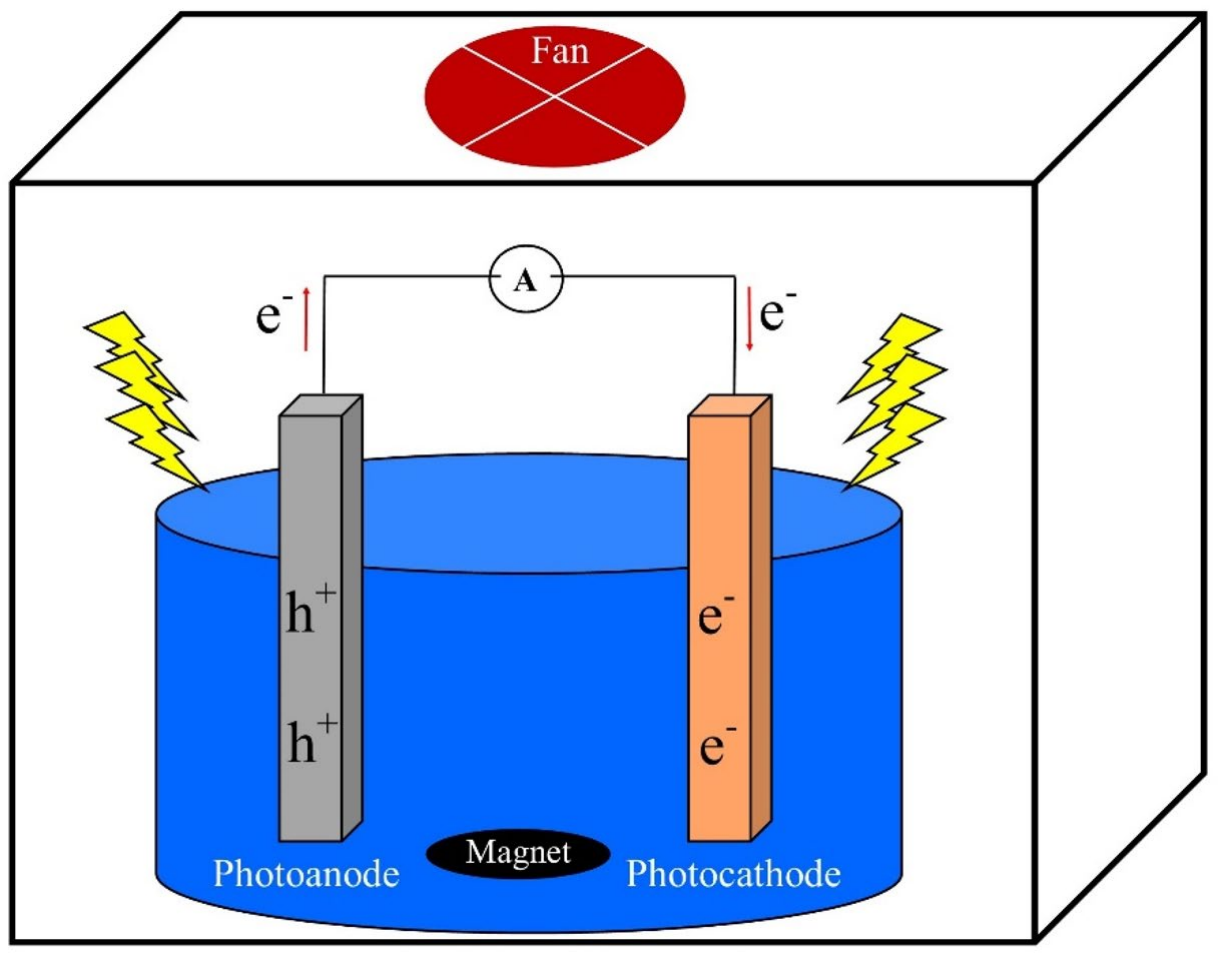
A schematic diagram for experimental set-up.

Photocatalytic degradation of SMZ is carried out using an immersed anode and cathode within a singular chamber, without the use of an ion-selective membrane, in a batch reactor. To keep the temperature constant at 25 ± 2 °C, the reactor chamber is cooled by a fan (Fig. 1). The surface of both the anode and cathode is 3 × 2 cm, and there is a distance of 3 cm between the two electrodes. A 300W xenon lamp was utilized as the light source. The SMZ antibiotic solution is stirred in the dark for a duration of 30 min to determine its absorbance. Subsequently, the stirring process is maintained under light conditions, and 2 ml of the solution is periodically extracted from the PFC system. The concentrations of SMZ were determined using a high-performance liquid chromatography-mass spectrometry (HPLC–MS) equipped with a C18 column (2.1 × 100 mm, 3.5 μm) at a wavelength of 270. The mobile phase consists of (A) methanol and (B) trichloroacetic acid in a ratio of 80:20. The degradation efficiency of SMZ is obtained from the following equation.

1 $$\mathrm{Degradation\; efficiency }\left(\mathrm{\%}\right)=\frac{{\text{C}}-{C}_{0}}{{C}_{0}} \times 100$$

The C_0_ and C represent the concentration and initial concentration at the specified time, respectively. The impact of the parameters, pollutant concentration (10–40 ppm), pH (3–9), and irradiation time (0–120 min) are evaluated.

The quantification of mineralization was assessed using a total organic carbon (TOC, Multi N/C, 3100, Germany) analyzer.

### Electrochemical tests

The PFC system utilizes a digital multimeter (specifically, the Victor-VC97 model) to measure voltage and current. The polarization curve is then plotted using the calculated data. Equation (2) is used to determine the generated power per unit of anode area. Additionally, the fill factor (FF), an important factor for energy conversion efficiency, can be calculated using Eq. (3).

2 $${\text{P}}={\text{V}}\times {\text{I}}$$

3 $$\mathrm{FF }=\frac{{{\text{P}}}_{{\text{max}}}}{{{\text{J}}}_{{\text{sc}}}\times {{\text{V}}}_{{\text{oc}}}}$$

In the equation, P_max_ denotes the real maximum power, J_sc_ corresponds to the short circuit current density, and V_oc_ represents the open circuit voltage^42^.

Photoelectrochemical measurements were performed on using a 25 mL three-electrode test system on an electrochemical workstation, consisting of a working electrode, counter electrode (platinum plate), and reference electrode (Ag/AgCl). TiO_2_/NiF, UiO-66-NH_2_-TiO_2_/NiF and pre-modified NiF were respectively used as working electrodes to determine the electrochemical characteristics. The photocurrent responses of the sample were assessed by measuring the amperometric curve (I-t) with a bias voltage of 0 V (open circuit voltage) for 400 s in Na_2_SO_4_ 0.5 mol/L electrolyte. Additionally, electrochemical impedance spectroscopy (EIS) was conducted on the synthesized materials in same condition electrolyte, at the open circuit potential within a frequency range of 0.1 to 100 kHz, both in the presence and absence of light.

## Results and discussion

### Photoanode characteristics

**Figure 2:**
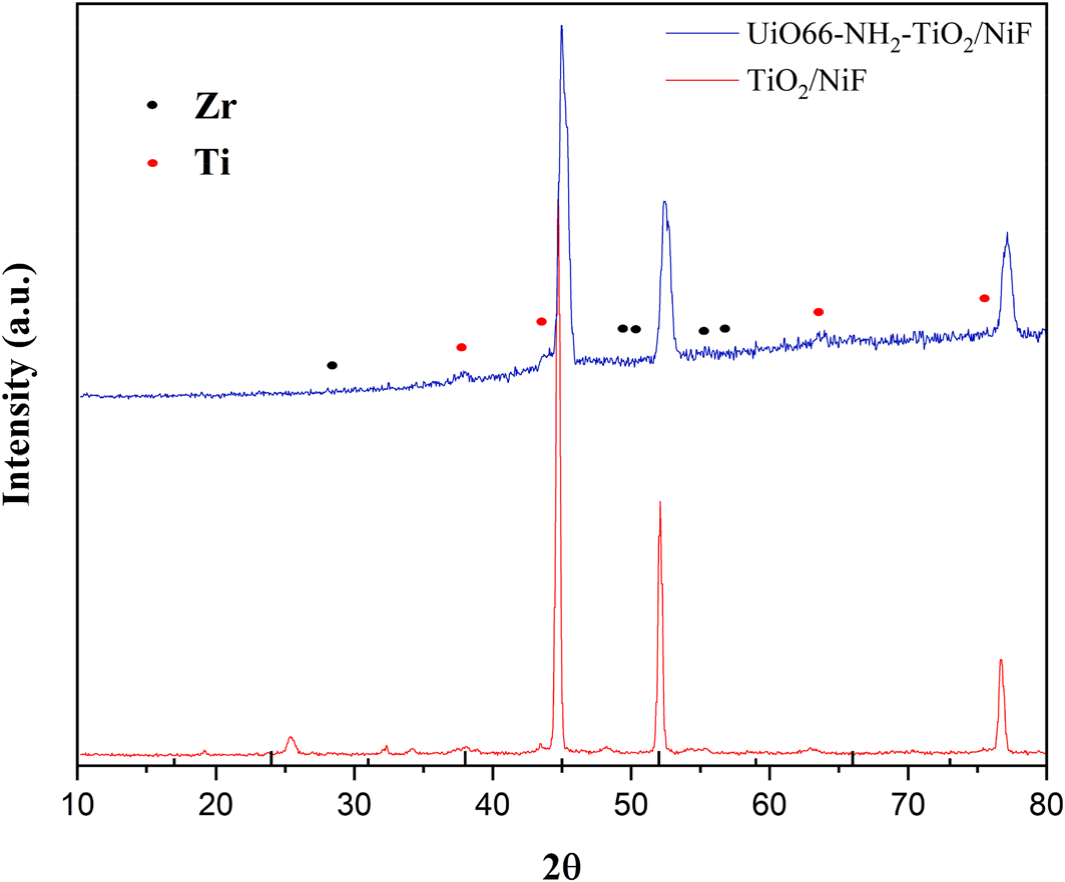
XRD patterns of TiO_2_/NiF and Uio-66-NH_2_-TiO_2_.

XRD was employed to investigate the crystal phase of the acquired products. The TiO_2_-NiF pattern exhibits in Fig. 2 seven major diffractions at 25.34°, 38.01°, 54.09°, 52.04°, 55.42°, 62.9°, 76.66° angles corresponded to the (101), (004), (105), (202), (211), (204), (301) crystal planes, respectively. In addition to TiO_2_-NiF peaks, the pattern of UiO-66-NH_2_-TiO_2_/NiF shows five major diffractions at 17.33°, 21.11°, 27.31°, 32.76°, 37.64° according to previous reports^43,44^. As is commonly understood, the inclusion of UiO-66-NH_2_ in UiO-66-NH_2_-TiO_2_/NiF has resulted in a reduction in the intensity of peaks associated with TiO_2_, indicating the effective loading of nanoparticles onto nickel foam. Furthermore, the peaks observed at 44.6°, 51.9°, and 76.4° were attributed to the presence of the Ni foam substrate.

**Figure 3:**
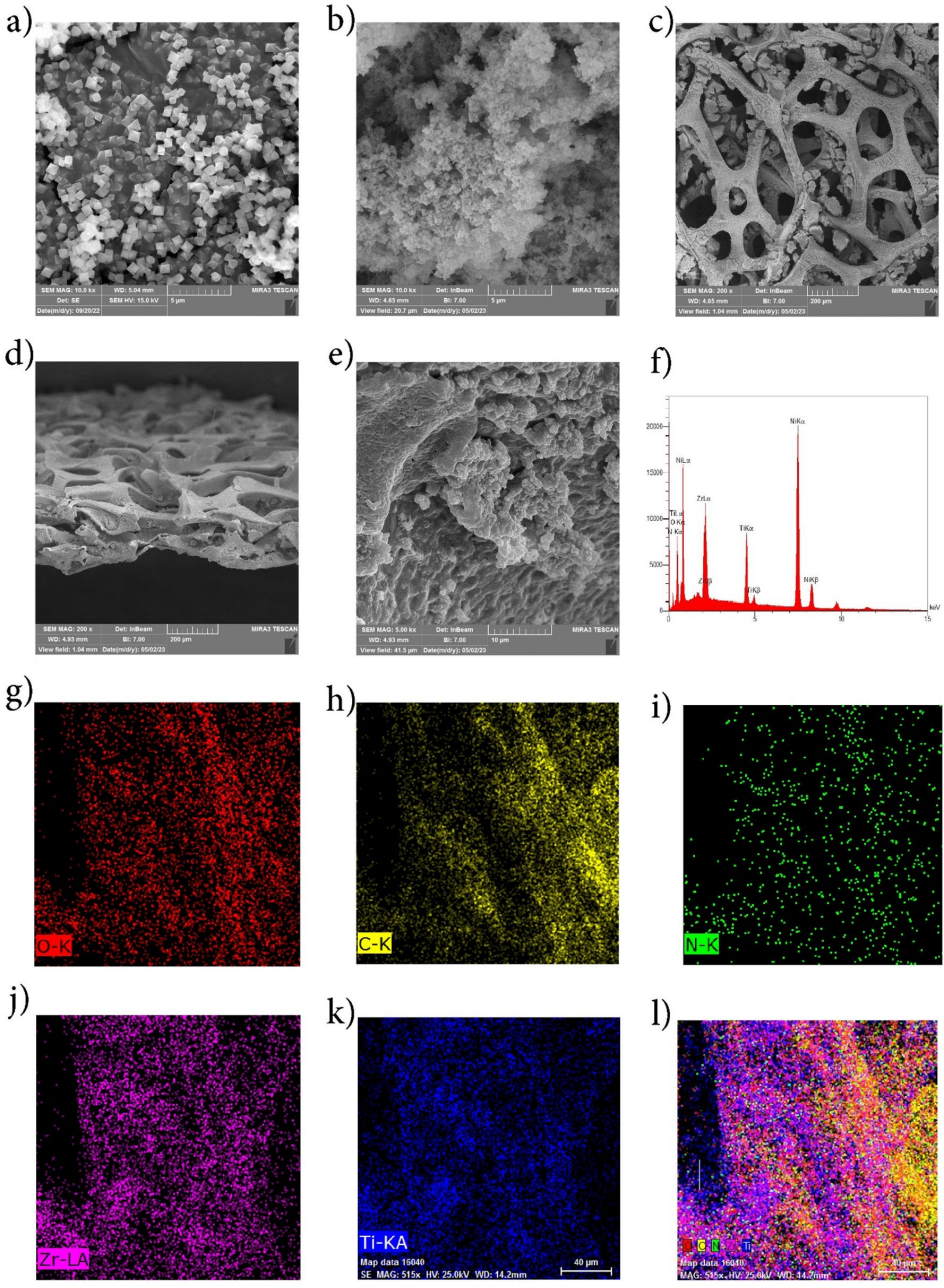
FE-SEM images of Uio-66-NH_2_ (**a**), Uio-66-NH_2_-TiO_2_ (**b**), Uio-66-NH_2_-TiO_2_/NiF (**c**), and cross-sectional image of Uio-66-NH_2_-TiO_2_/NiF (**d**,**e**), EDX spectra (**f**), and elemental visualization Zr, N, Ti, O, C, (**g**–**l**) of photoanode.

The surface morphology, size and distribution of Uio-66-NH2-TiO_2_ catalyst on nickel foam were characterized by Fe-SEM technique. As depicted in Fig. 3a, UiO-66-NH_2_ shows a sleek and symmetrical octahedral morphology with sharp edges. The average side size is approximately 50 nm, which is completely agglomerated with TiO_2_ with spherical structure (Agglomerate ball structure) is completely covered (Fig. 3b), which provides more active sites for radiation and contaminant contact. The accumulation of titanium catalyst nanoparticles is attributed to their high surface-to-volume ratio. The provided information indicates that the nanoparticles exhibit a spherical and nearly uniform shape, with an estimated size of approximately 25 nm. Mansouri et al. also obtained similar results in their study with nanoparticles size of approximately 28 nm and showed the stabilization of titania particles uniformly on the synthesized MOF as a support^45^. Figure 3c Fe-SEM shows the successful immobilization of UiO-66-NH_2_/TiO_2_ photocatalyst on nickel foam. The crystals have an approximate crystal size in the range of 41–60 nm. Figure 3d and e shows a cross-sectional view of Uio-66-NH_2_-TiO_2_/Ni Foam, it can be concluded that after coating, the hollow structure in the nickel foam is significantly filled. The unique microstructure topography and interconnected cell structure of nickel foam contribute to a notably expansive surface area. This characteristic aids in effectively immobilizing the catalyst within the foam nickel, rendering it versatile for various applications. Visual examination reveals an uneven surface on the catalyst sheet, presumed to optimize light reflection and enhance its potential for multiple light-utilizing functions. Furthermore, the presence of numerous gaps between the catalyst and nickel foam is anticipated to facilitate efficient transfer, absorption, and subsequent reaction processes^36,37^. Also, the visualization spectrum of elements is shown in Fig. 3g–l.

**Figure 4:**
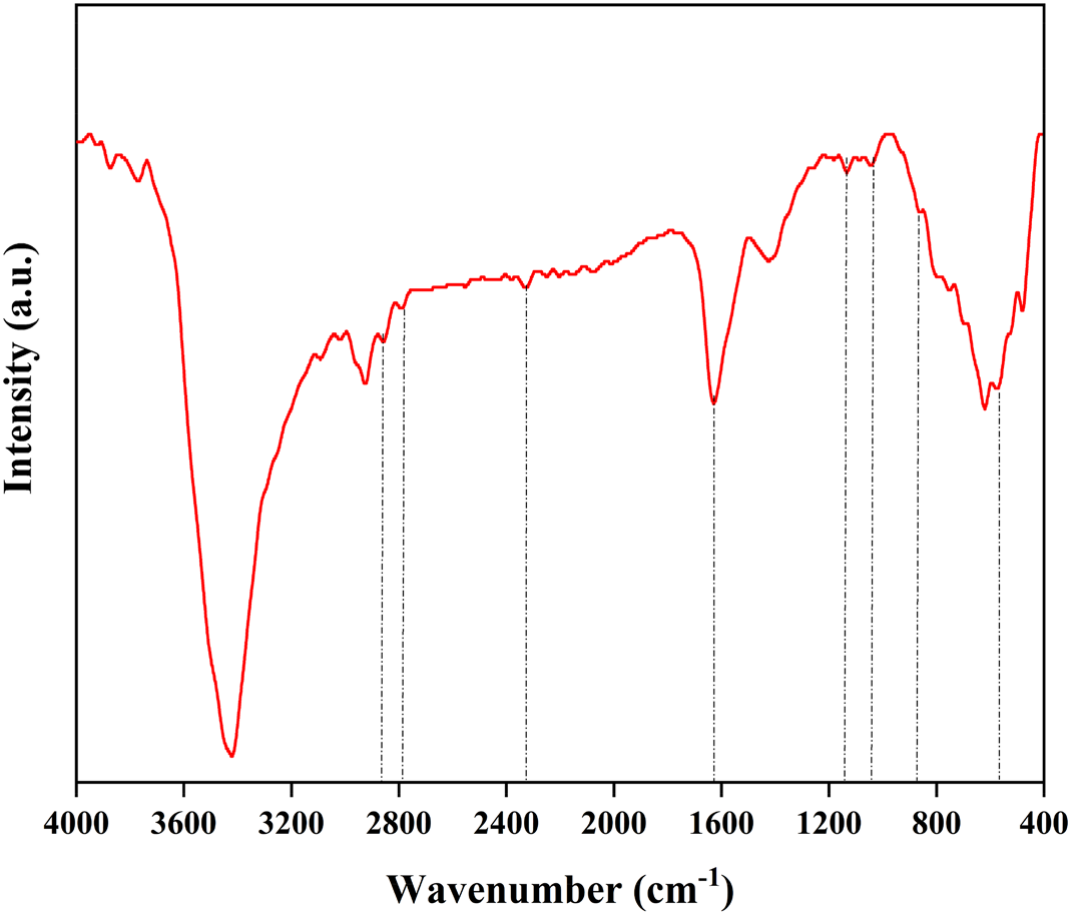
FT-IR of UiO-66-NH_2_/TiO_2_.

Figure 4 illustrates the FT-IR outcomes of UiO-66-NH_2_/TiO_2_. Bands ~ 575 and 620 cm^−1^ indicate tensile vibrations of Ti-O^46^. The peak observed at 1044 cm^−1^ can also be attributed to the existence of carboxylate groups (COO−) within the organic linkages of MOFs^47^. The confirmation of the amine group's presence in the MOF structure is indicated by the sharp stretching vibrations observed at 16,281 cm^−1^, which correspond to the N–H bond of the primary amine^48^. The frequency bands at 3421, 2791, and 2857 corresponded to the bending vibrations of O–H and C–H bonds^44^. The OH bond in water molecules can also be identified by the 3421 cm^−1^ band, which corresponds to its stretching vibrations. Aromatic bands (C=C) can be observed at 2328 cm^−1^. The stretching of the C-N band at 1133 cm^−1^ is also evident for the 2-aminoterephthalate ligand. UiO-66-NH_2_ exhibits other characteristic vibrations such as the C–O band at 1044 cm^−1^ and the out-of-plane N–H motion at 861 cm^−1^^48–50^.

**Figure 5:**
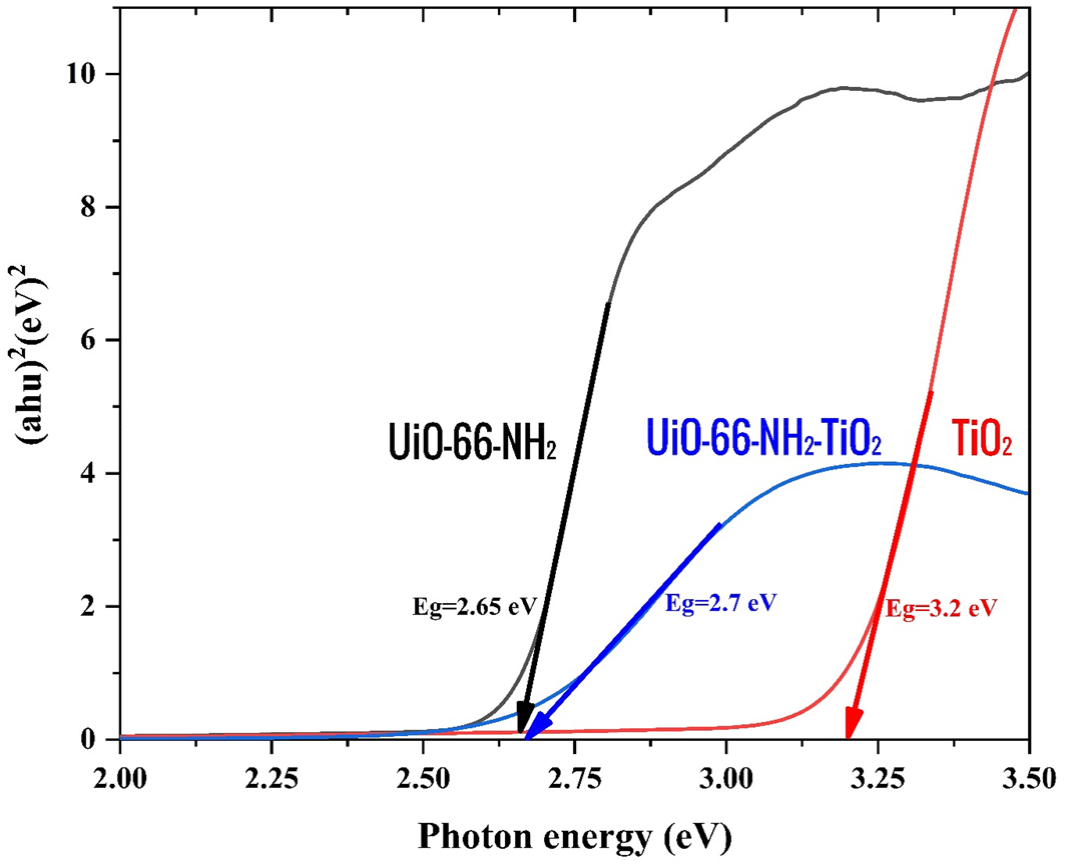
Estimated band gaps for UiO-66-NH_2_, TiO_2_, and UiO-66-NH_2_/TiO_2_.

To assess the optical response range and explore the interaction between the two components in the catalyst material, a UV–visible emission reflectance spectrum (DRS) was employed. According to the information obtained, the estimated band gaps (Fig. 5) for UiO-66-NH_2_ and TiO_2_/Ni nanoparticles are approximately 2.65 eV, 3.21 eV respectively. The UiO-66-NH_2_/TiO_2_ composite exhibited the expected inheritance of the optical property from UiO-66-NH_2_. This was evident as the band gap of the UiO-66-NH_2_/TiO_2_, (2.69 eV) nanocomposite was lower compared to that of TiO_2_ alone, resulting in an enhanced optical response that extended into the visible region^43^. Additionally, this improvement in optical response helped to prevent electron–hole recombination, thereby enhancing the photocatalytic performance^44^.

### Photoelectrochemical teste

#### Chronoamperometry

**Figure 6:**
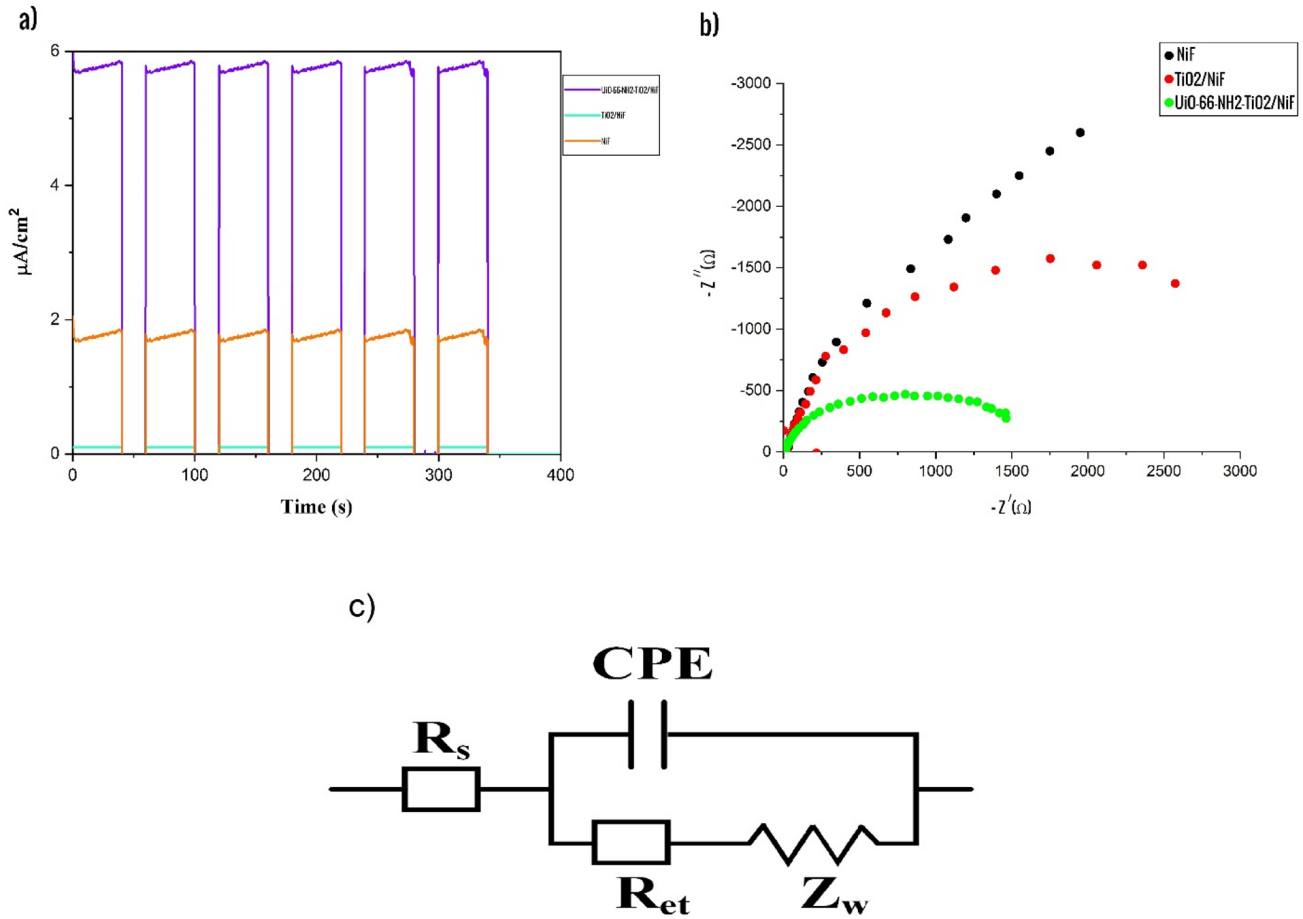
Chronoamperometry curve (I-t) (**a**), EIS plot (**b**), equivalent circuit used in fitting the data (**c**).

The Fig. 6a displays the I-t curves for the electrodes under investigation, indicating the extent of charge transfer during periodic light on/off cycles when the potential was adjusted to 0 V relative to the Ag/AgCl electrode. Pure NiF has minimal impact on light response, but the inclusion of TiO_2_ in NiF exhibits a significant effect. UiO-66-NH_2_-TiO_2_/NiF composites demonstrate notable and consistent photocurrent responses, suggesting efficient charge separation within the composite materials containing TiO_2_/NiF. This could be attributed to the enhanced electrical conductivity of nickel foam resulting from the reduced resistance following the deposition of MOF nanoparticles^36^. As a result, UiO-66-NH_2_-TiO_2_/NiF exhibits a rapid and stable optical current generation of 6 μA/cm^2^, whereas NiF only achieves approximately 0 μA/cm^2^ in terms of optical current generation. Furthermore, extending the reaction time beyond 400 s did not suppress the intensity of the light current, suggesting that they enhance consistent photoelectric activity. The enhanced conversion of photoelectrons can be attributed to the improved segregation of photo-excited electrons and holes, which can potentially be ascribed to the well-matched band structure and high conductivity of nickel foam. The employment of Ni as a dopant results in a reduction in the band gap. At the same time, the porous characteristics of the photocatalyst trap light effectively and provide a significant contact surface area^35,51,52^.

#### Electrochemical impedance spectroscopy

The Fig. 6b demonstrates the application of Electrochemical Impedance Spectroscopy (EIS) for assessing the efficiency of electron–hole recombination. In EIS measurements, the electron transfer resistance (R_ct_), also known as charge transfer resistance, can be analyzed using Nyquist diagrams. This resistance is represented by the diameter of a semicircle in the diagram. A smaller arc radius in EIS indicates lower resistance to charge transfer and less inhibition of recombination^53,54^. As shown in Fig. 6b, the amount of R_ct_ is shown for UiO-66-NH_2_-TiO_2_/NiF and TiO_2_/NiF. The equivalent circuit model fitted to the impedance data that its components are Ret, the resistance of the electron transfer between the solution and the electrode surface; Z_W_, the Warburg element; CPE, the constant phase element, and Rs, the solution resistance (Fig. 6c). In the case of UiO-66-NH_2_-TiO_2_/NiF, the smallest observed arc radius corresponds to a reduced resistance against charge transfer. This observation suggests that the deposition of the nanocatalyst has resulted in a decrease in transfer resistance^55^. UiO-66-NH_2_-TiO_2_/NiF can greatly enhance the separation and transfer of electron–hole (e^−^/h^+^) pairs, thereby increasing photocatalytic activity. In the PFC system, the UiO-66-NH_2_-TiO_2_/NiF photoanode, supported by a porous nickel foam substrate, plays a crucial role in generating photo-excited electron–hole pairs and enhancing electrical energy output^37,56^.

### The impact of process parameters on SMZ degradation in the PFC system

#### The impact of SMZ concentration

**Figure 7:**
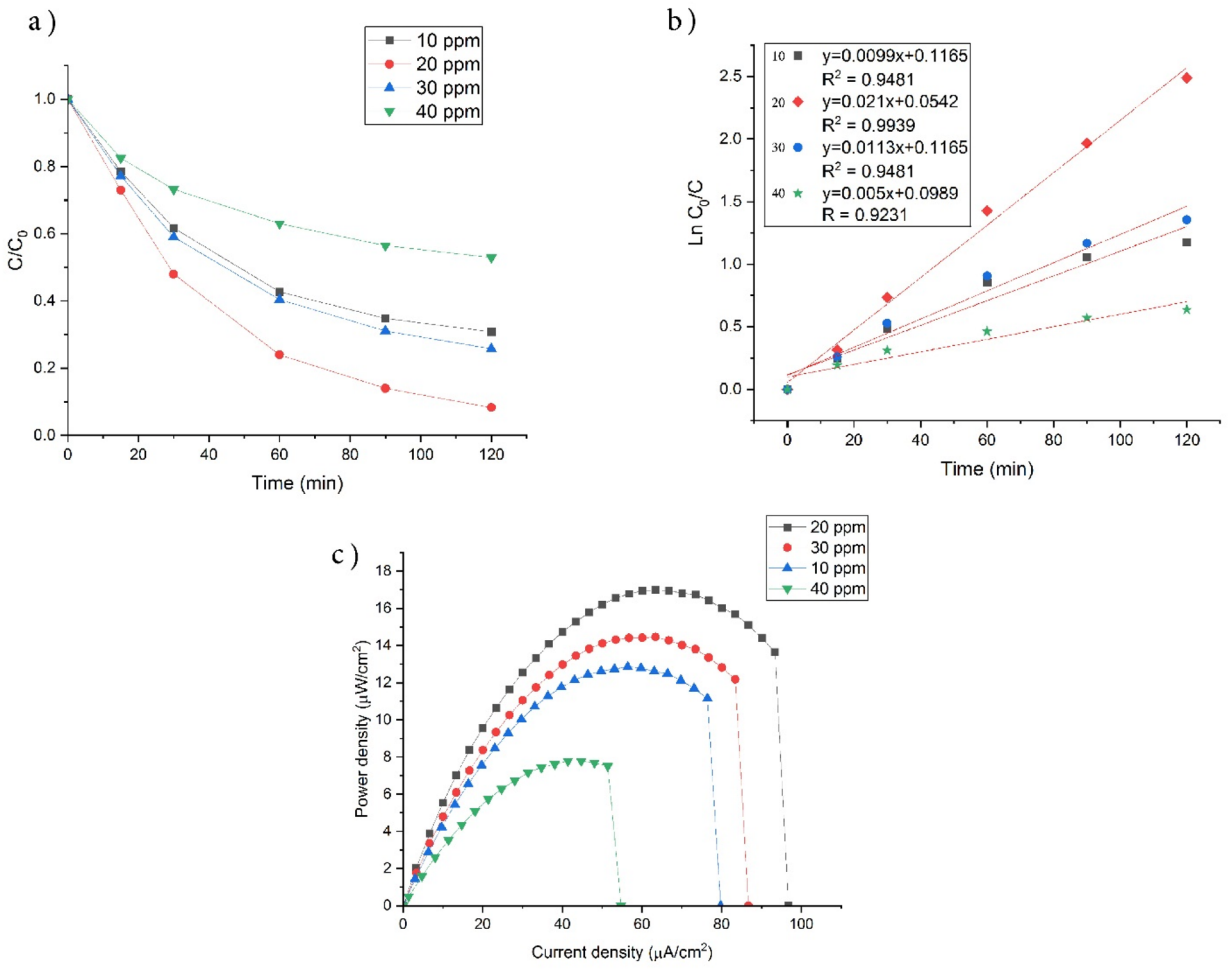
PFC efficiency: (**a**) SMZ degradation efficiency; (**b**) kinetic photocatalytic degradation; (**c**) power density curves of UiO-66-NH_2_-TiO_2_/NiF photoanode at different initial SMZ concentration in PFC.

As illustrated in Fig. 7a, increasing the concentration of SMZ from 10 to 20 resulted in an increase in efficiency from 69.2 to 91.7%. However, when the concentration was further raised 40 ppm, the efficiency decreased to 47.1%.

When the antibiotic concentration is low (10 ppm), the byproducts generated alongside the pollutant compete for the active groups involved in the degradation process, leading to a decrease in the removal rate^13,57^. In general, the formation of hydroxyl radicals is the rate-determining step in the reaction for pollutant removal. This is because hydroxyl radicals react rapidly with pollutants. OH radicals are generated through the reaction of holes with OH^**−**^ and adsorbed H_2_O. However, if the adsorbed OH positions are occupied by contaminant ions, the production of OH radical’s decreases. As a result, the presence of pollutants and their oxidation intermediates negatively impacts the efficiency of pollutant removal by covering the active sites of the photocatalyst^58^.

Figure 7b shows the photocatalytic degradation under different concentrations of SMZ using first order kinetic model. The reduction in removal efficiency and K value at high concentrations (30 and 40 ppm) may be attributed to the adsorption of pollutant molecules on the electrode surface and the active surface of the photocatalyst deposited on the nickel foam. This leads to a decrease in active sites and a delay in light penetration, as reported in references^59,60^.

Also, the power density curve (P-I) in the Fig. 7c demonstrates that the maximum power was achieved at a concentration of 20 ppm. As the pollutant concentration increases, the scavenging of generated h^+^ also increases, leading to an increase in the separation of h^+^/e^−^ and facilitating more electronic transport to the cathode. Consequently, amplifies energy generation. However, the initial concentration surpasses a certain threshold, the SMZ molecules present in the solution reduce the light absorbed by the photocatalyst. Consequently, the photoexcitation of electrons in the photoanode becomes limited, resulting in lower electricity generation compared to the 20 ppm concentration. Moreover, at 40 mg/L, there may be a decrease in the spontaneous of electrons towards the cathode, which can reduce the production of superoxide radicals (% O_2_^−^). These radicals are classified as reactive oxygen species (ROS) and possess the ability to efficiently degrade various types of organic compounds^61,62^.

#### The impact of pH

The pH of the initial solution plays a crucial role in determining catalyst surface charge factors (active sites) and the properties of organic pollutants. The impact of pH was examined within the pH range of 3.0 to 9.0, by adjusting with 1M HCl and/or NaOH. In different pH conditions, sulfonamides have cationic, zwitterionic and anionic forms^63^. Based on the pK_a_ values, SMZ exists predominantly in positively and negatively charged forms at pH values lower than pK_a1_ and above pK_a2_, respectively. However, between pK_a1_ and pK_a2_, SMZ is primarily present in its neutral form. Since the catalyst has less impact on cationic and anionic species, the highest efficiency was achieved at pH 6^64^.

**Figure 8:**
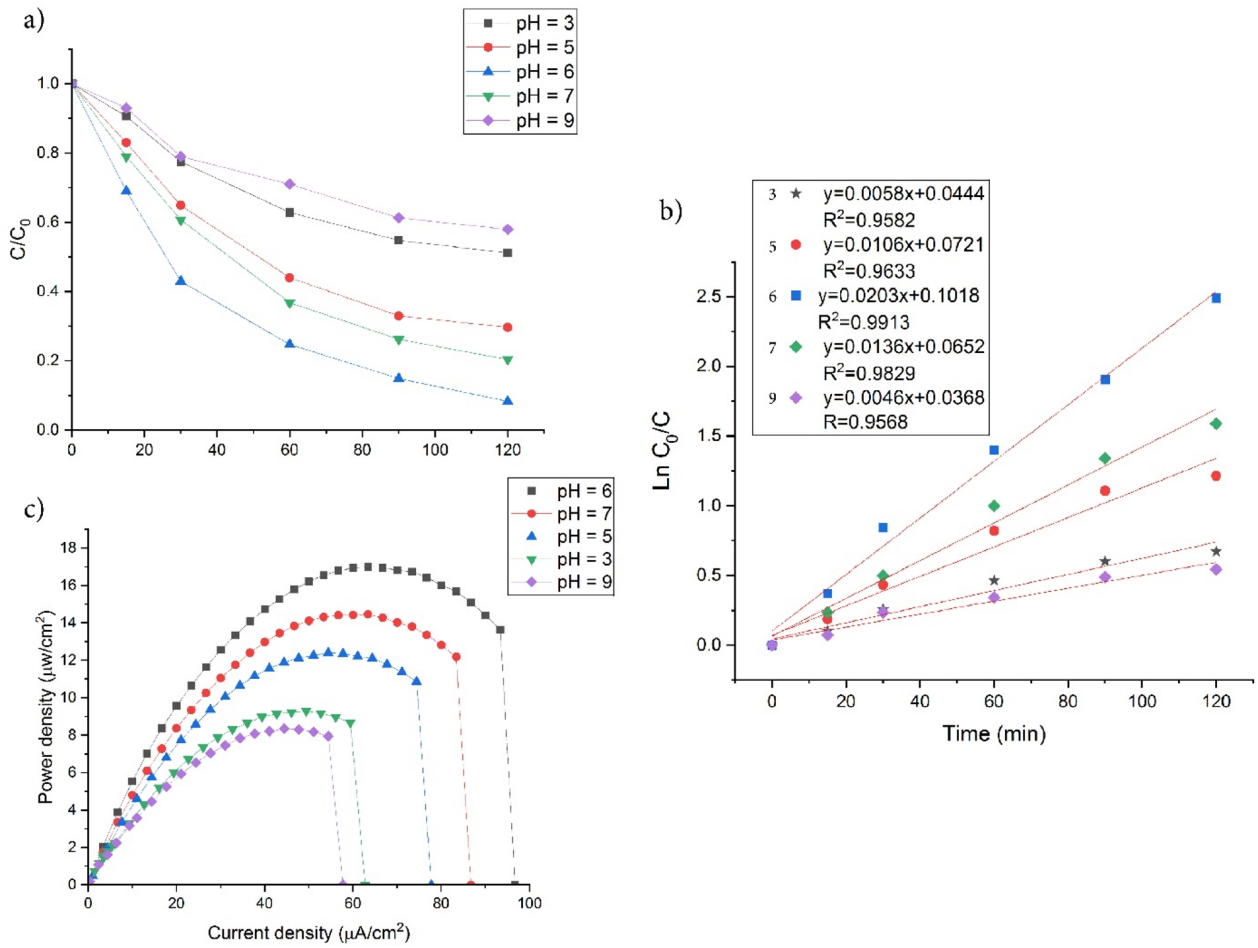
PFC efficiency: (**a**) SMZ degradation efficiency; (**b**) kinetic photocatalytic degradation; (**c**) power density curves of UiO66-NH_2_-TiO_2_/NiF photoanode, at different pH with 20 ppm SMZ in PFC.

The UiO-66-NH_2_ surface in the pH acidic exhibited a positive charge as a result of the protonation of the amino group. This positive charge led to electrostatic repulsion with cation SMZ ions, thereby resulting in relatively low removal efficiency under acidic conditions. As the pH increases, the amino groups on UiO-66-NH_2_ become neutralized^65^. In acidic conditions, there is a higher rate of recombination of electron–hole pairs in the photoanode because of the lower presence of hydroxyl groups in the solution. It is believed that the percentage of hydroxyl groups (% OH) plays a significant role in breaking down the N = N^−^ conjugated system in sulfonamide compounds^66^. At lower pH conditions, a significant number of H^+^ ions have a tendency to interact with the azo bond, thereby decreasing the electron density of the azo group and directly impacting the output power and efficiency of the PFC (Fig. 8a–c). When the pH level was elevated, the catalyst's negative surface repelled the negative charge of SMZ anionic forms and hindered the oxidation of pollutants in the photoanode. Nevertheless, proton reduction can take place in alkaline conditions, leading to the inhibition of the cathodic oxygen reduction reaction (ORR)^67–69^.

#### The impact of irradiation time

Increasing the duration led to an increase in Chances for the photoanode and photocathode to generate a greater number of active radicals and eliminate SMZ^70^. As the reaction time extended from 30 to 90 min, the efficiency exhibited a steep slope and surpassed 85%. With the reaction proceeding for up to 120 min, the efficiency slope became more gradual and reached to 91.7%.

#### The impact of light intensity

**Figure 9:**
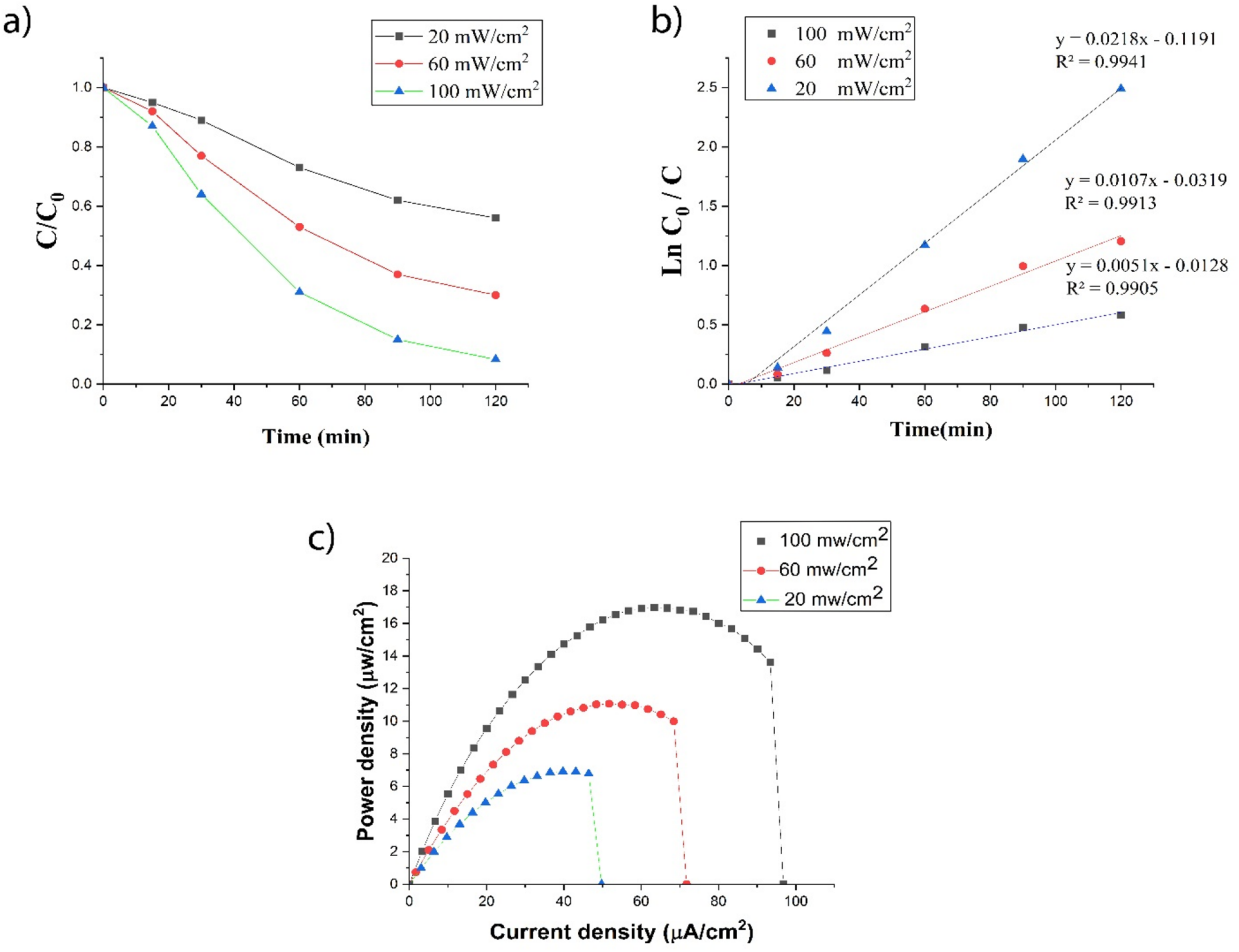
PFC efficiency: (**a**) SMZ degradation efficiency; (**b**) kinetic photocatalytic degradation; (**c**) power density curves of UiO-66-NH_2_-TiO_2_/NiF photoanode, at different light intensity with 20 ppm SMZ in PFC.

In Fig. 9a–c, the results of radiation intensity changes on the efficiency of pollutant removal (a), simultaneous energy production (b) and kinetic photocatalytic degradation (c) are shown. As it can be seen from the figure, the removal efficiency increased from 42 to 91.7% for the pollutants on average with increasing the radiation intensity from 20 to 100 mW/cm^2^. Increasing the radiation intensity led to the absorption of photons by the anode and the production of more electron holes. On the other hand, the generated electron moves towards the photocathode and superoxide radical is generated, which increases the efficiency. Also, with the increase of light intensity from 20 to 100 mW/cm, the maximum power density of PFC reached from 6.91 µW/cm to 16.98 µW/cm^2^ because with the increase of light intensity, the speed of photoelectrochemical reaction in the photoanode increases. With stronger light irradiation, more excited electron–hole pairs were created in the photoanode and the oxidation process of organic substances was strengthened and more electrons were produced and transferred to the cathode. Hence, the performance of the developed PFC increased significantly with increasing light intensity^71–73^.

### Optimization and performance of PFC system

The optimization is done by the method of one factor in time to find the maximum light degradation efficiency. Under optimal condition ((SMZ concentration = 20 ppm, pH = 6, radiation time = 120), the UiO-66-NH_2_-TiO_2_/NiF composite achieved a performance of 91.7%, while the TiO_2_/NiF composite reached 56% (Fig. 11a). This improvement can be attributed to narrower band gaps and reduced recombination, as indicated by UV–vis DRS analysis. The transfer of electrons from the anode to the cathode can result in an elevation of the superoxide radical within the cathode. Additionally, the excellent electrical conductivity of nickel foam, serving as a substrate, facilitates the efficient separation of hole electrons and reduces recombination.

**Figure 10:**
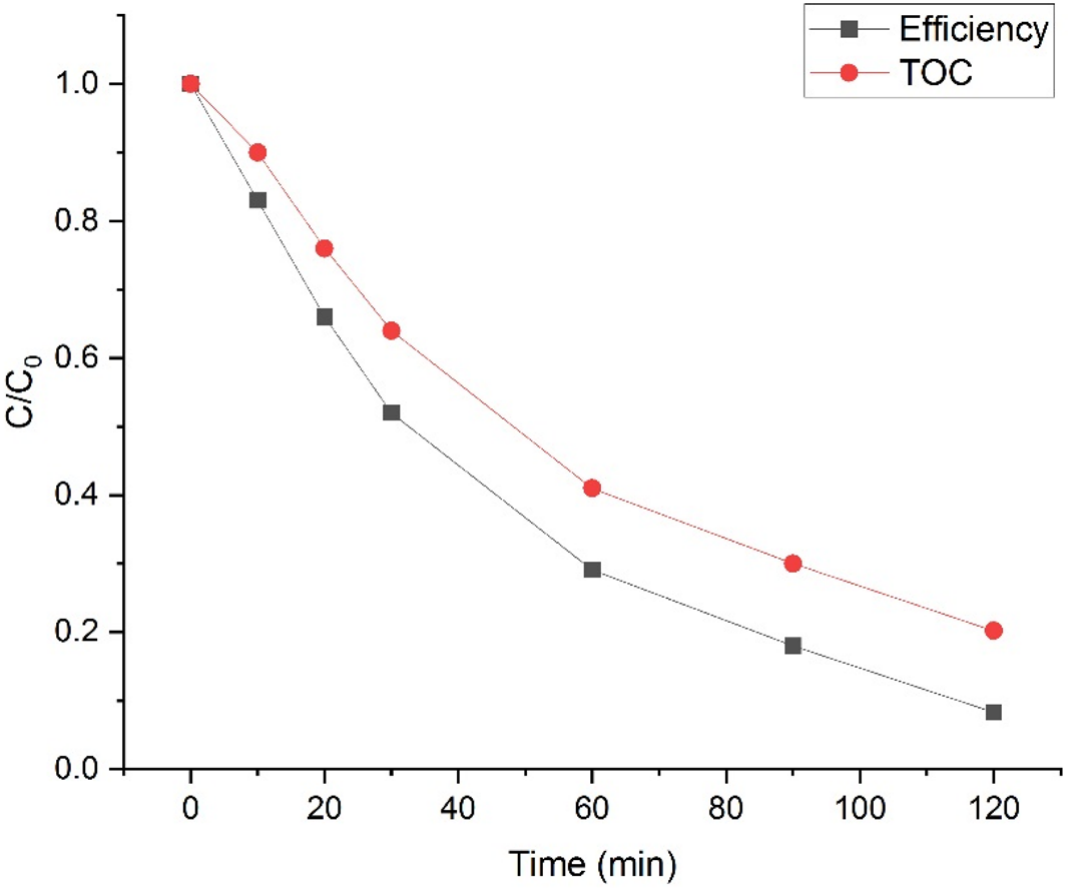
Analysis of total organic carbon (TOC) under optimal conditions.

The analysis of TOC (Fig. 10) under optimal conditions for mineralization resulted in a value of 81.4%, which is lower compared to the SMZ removal value. This decrease may be attributed to the generation of intermediate compounds, necessitating additional time for full mineralization^61^.

**Figure 11:**
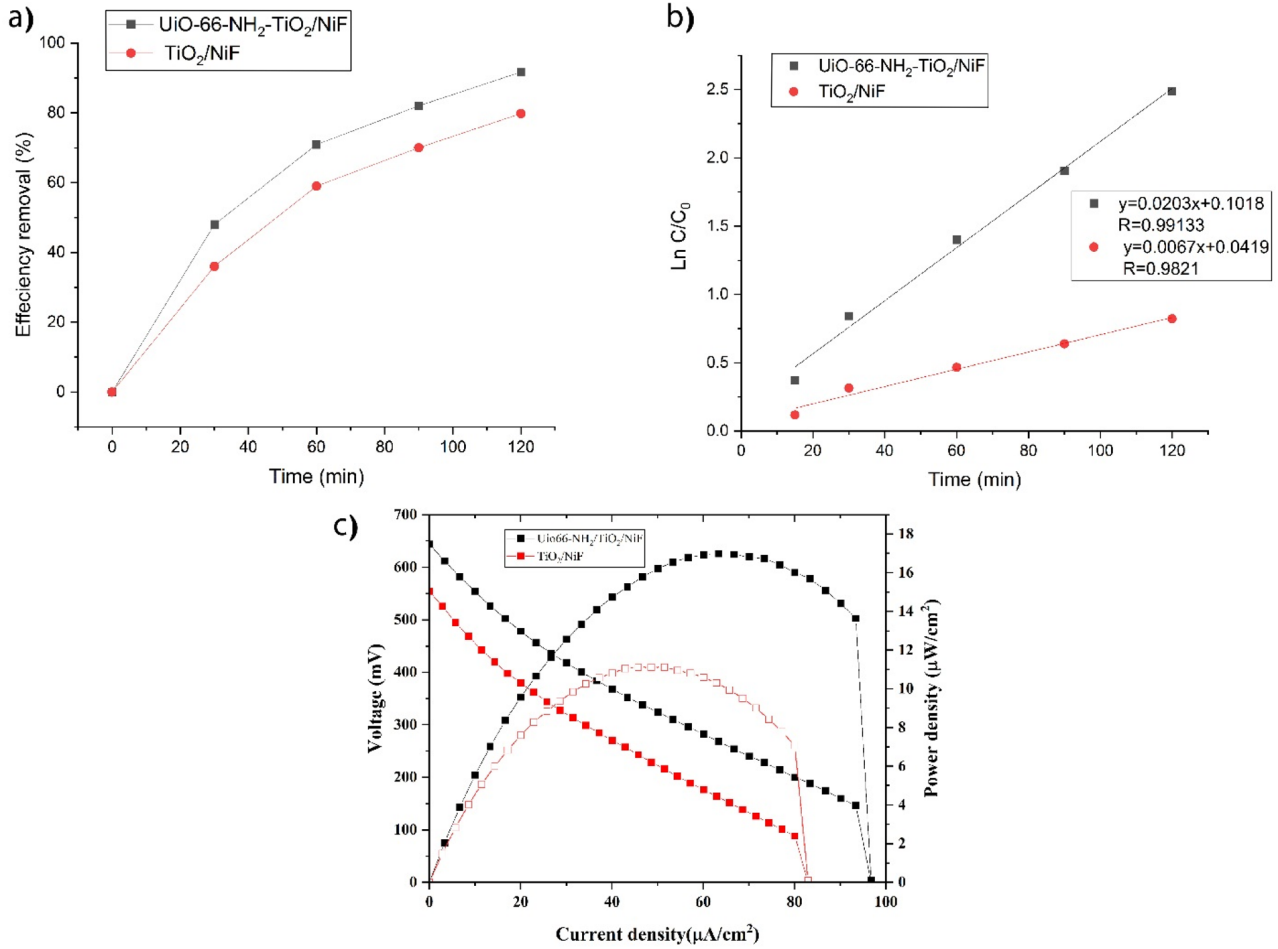
PFC efficiency: (**a**) SMZ removal efficiency; (**b**) kinetic photocatalytic degradation, (**c**) polarization (J-V) and power density (J-P) curves (**c**) for different photoanode in optimal condition.

The rate constant (k) for a first-order reaction can be determined by using the equation Ln(C_0_/C) = kt. The Fig. 11b shows the degradation kinetics of SMZ during xenon irradiation, which clearly follows a first-order reaction.

To further investigate the photoanode activity, polarization (J-V) and power density (J-P) curves has been drawn (Fig. 11c). The results reveal that the photocurrent response of UiO-66-NH_2_-TiO_2_/Ni is three times higher than that of TiO_2_/Ni. This significant increase can be attributed to the efficient separation and transfer electrons.

In order to assess the efficiency of converting light energy into electrical energy in photocatalytic reactions, the metric utilized is photoelectric conversion efficiency. This is predominantly characterized by two primary criteria: (a) Incident Photon-to-Current Efficiency (IPCE): IPCE gauges the ratio of photocurrent to incident photons, offering insights into the effectiveness of electron transport processes during photoabsorption^74^. The calculation is based on formula (4):

4 $$\mathrm{IPCE }=\frac{1240 \times {{\text{J}}}_{{\text{SC}}}} {\lambda \times {\text{P}}}\times 100\%$$

(b) Chemical-to-Electricity Conversion Efficiency (η): This parameter appraises the overall efficiency of transforming chemical energy generated through photocatalysis into electricity^75^. The calculation is based on formula (5):

5 $$\upeta = \frac{{{{\text{J}}}_{{\text{sc}}} \times {{\text{V}}}_{{\text{oc}}} \times \mathrm{ FF}}}{{\text{P}}} \times 100\%$$

where λ represents the light wavelength, and P denotes the light power intensity. In optimal conditions, the values for IPCE and η are achieved at 2.611% and 0.177%, respectively.

For the best conditions, the following values are obtained for UiO-66-NH_2_-TiO_2_/NiF: P_max_ = 16.98 μW/cm^2^, J_sc_ = 96.75 μA/cm^2^, V_oc_ = 644 mV, and FF = 0.271. For TiO_2_/NiF, the values are P_max_ = 6.48 μW/cm^2^, J_sc_ = 6.75 μA/cm^2^, V_oc_ = 554 mV, and FF = 0.24.

### Reusability and stability of photoanode

**Figure 12:**
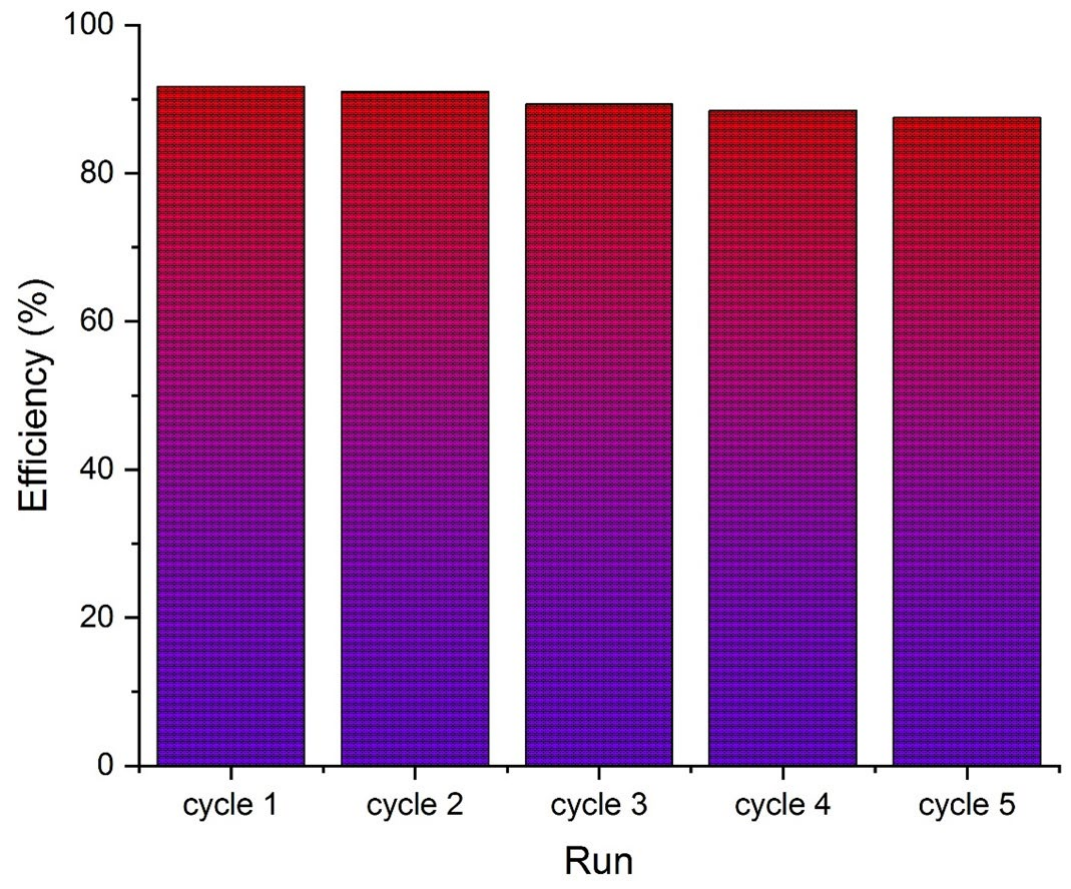
The stability of the photoanode after five cycles.

One of the influential factors in PFC systems is the stability of the photoanode. The reusability of the UiO66-NH_2_-TiO_2_/NiF anode is assessed for removal efficiency and electrochemistry after five cycles, each lasting 120 min. After 120 min of reaction, the photoelectrodes were withdrawn from the reaction environment and washed with distilled water. The photoelectrodes were dried in an oven at 90 °C and then added to fresh electrolyte for subsequent cycles. The photodegradation of SMZ exhibited a decrease from 91.7 to 88 (approximately 3.7%) after five cycles, which can be attributed to photon corrosion (Fig. 12).

The findings indicated that the catalyst incorporated into the photoanode exhibits favorable photocatalytic properties within the PFC system. Also during the initial cycle, J_sc_, V_oc_, and P_max_ were measured at 16.98 μW/cm^2^, 96.75 μA/cm^2^, and 644 mV, respectively. However, in the fifth cycle, these values slightly declined to 14.92 μW/cm^2^, 92.51 μA/cm^2^, and 600 mV, respectively. This decrease in electrochemical performance can be attributed to the absorption of intermediate products, which subsequently elevate the resistance of the photoanode^76^.

Furthermore, the PFC system exhibited a photoanode mass loss rate of approximately 6%, providing further evidence of its long-term stability and excellent recyclability. This indicates that the developed PFC system not only demonstrated high photoelectric activity but also exhibited commendable stability.

### Mechanisms

**Figure 13:**
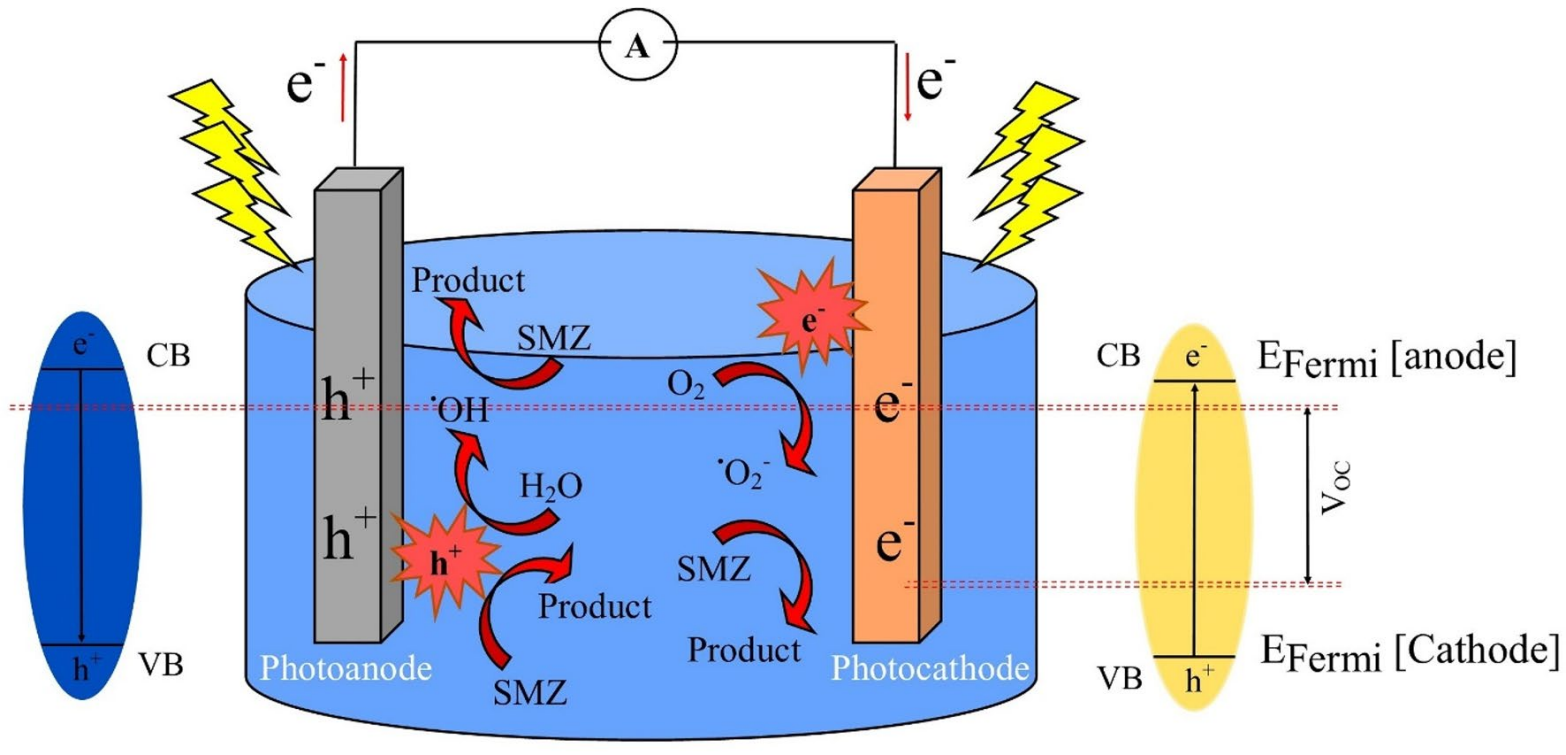
Schematic diagram of reactions occurred during photocatalytic process in PFC system.

The degradation process of SMZ in the PFC system initiates with the exposure of light to the photocatalyst in both the photoanode and photocathode, resulting in the generation of excited e^−^ and h^+^ at the conduction band (CB) and valence band (VB) edges, respectively (Fig. 13). The photoanode generates holes that can either eliminate pollutants or react with H_2_O to produce OH radicals. These radicals act as powerful oxidizing agents, capable of indiscriminately breaking down organic substances into harmless end products. Excited electrons within the photoanode are transported to the photocathode through the external circuit, thereby minimizing electron–hole recombination. Electrons within the photocathode have the ability to initiate the oxygen reduction reaction (ORR), resulting in the generation of superoxide radicals (O_2_^−⋅^). These superoxide radicals can subsequently undergo reactions with electrons (e^−^) and hydrogen ions (H^+^) to yield hydrogen peroxide (H_2_O_2_), as illustrated by the following equations:

6 $${\text{UiO66-NH}}_{{2}}{\text{-TiO}}_{{2}} /{\text{NiF}} + \, \left( {{\text{hv}}} \right) \, \to {\text{ h}}^{ + } + {\text{ e}}^{ - }$$

7 $${\text{h}}^{ + } + {\text{ H}}_{{2}} {\text{O }} \to {}^{ \cdot }{\text{OH }} + {\text{ H}}^{ + }$$

8 $${\text{e}}^{ - } + {\text{ O}}_{{2}} \to {\text{ O}}_{{2}}{^{ - \cdot }}$$

9 $${\text{O}}_{{2}}{^{ - \cdot }} + {\text{ H}}_{{2}} {\text{O }} \to {}^{ \cdot }{\text{OH }} + {\text{ OH}}^{ - }$$

10 $${\text{O}}_{{2}}{^{ - \cdot } }+ {\text{ 2H}}^{ + } + {\text{ e}}^{ - } \to {\text{ H}}_{{2}} {\text{O}}_{{2}}$$

11 $${\text{H}}_{{2}} {\text{O}}_{{2}} \to { 2}{}^{ \cdot }{\text{OH}}$$

12 $$\left( {{\text{h}}^{ + } + {}^{ \cdot }{\text{OH }} + {\text{ O}}_{{2}}{^{ - \cdot } }} \right) \, + {\text{ SMZ }} \to {\text{ CO}}_{{2}} + {\text{ H}}_{{2}} {\text{O }} + {\text{ other products}}$$

**Figure 14:**
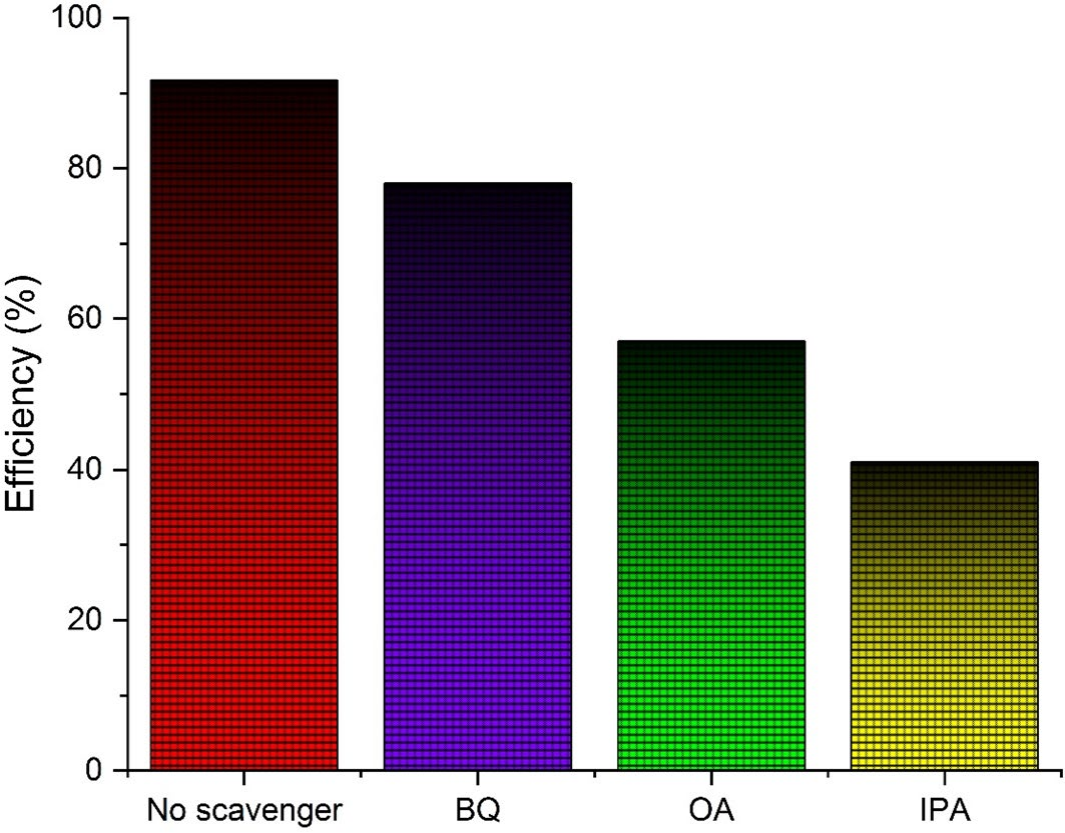
Active radical’s scavenger.

In order to clarify the role of UiO-66-NH_2_-TiO_2_/NiF in the charge transfer process, the radical trapping experiment with isopropanol (IPA), ammonium oxalate (OA), benzoquinone (BQ) respectively for OH^−^, ^+^h, O^−^_2_ radical were used^77^. As shown in Fig. 14, the addition of BQ was less effective in pollutant degradation, while the addition of IPA or OA greatly suppressed the reaction. Therefore, OH and h^+^ radicals may be considered as the main function in pollutant destruction.

#### Possible pathway of SMZ degradation

**Figure 15:**
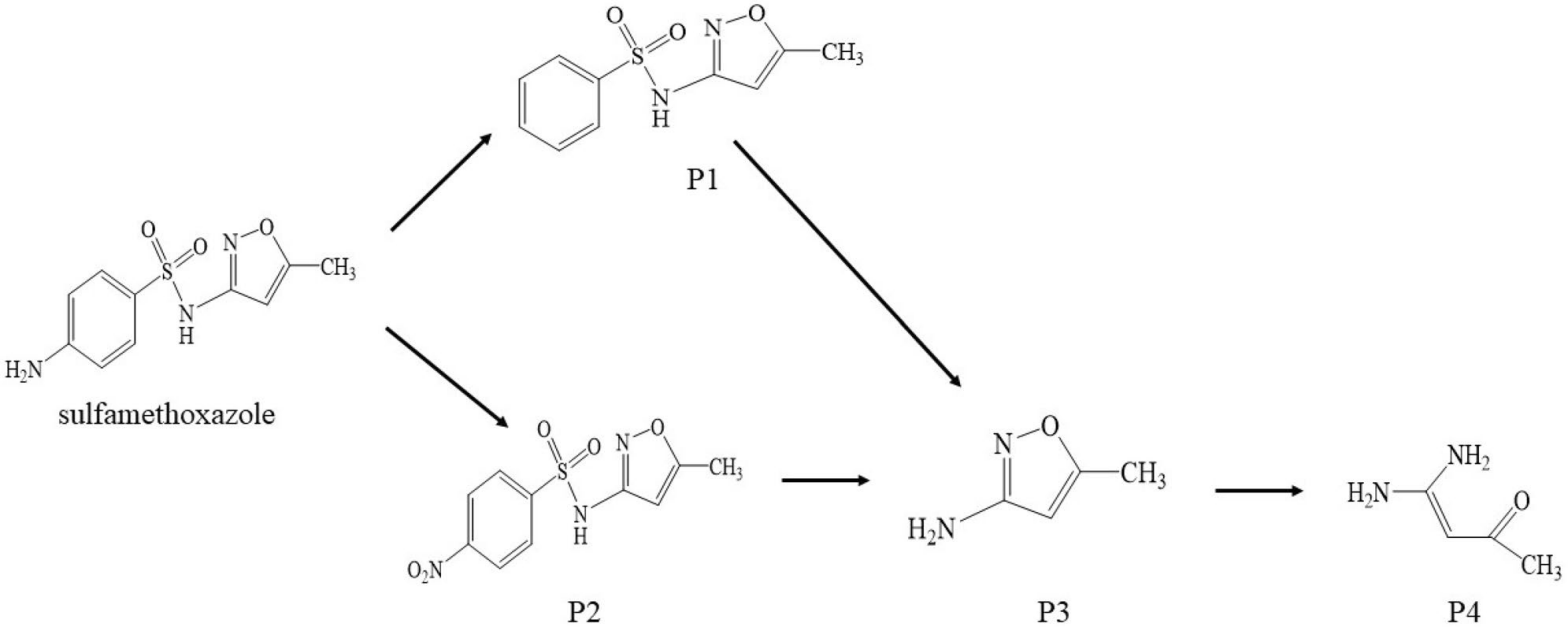
Proposed degradation pathways for SMZ in the PFC system.

Based on the intermediates were identified, possible degradation pathways of SMZ in the PFC system are proposed in Fig. 15. It is assumed that the deamination of the benzene ring in SMZ may result in the generation of P1. The attack of ROS and extraction of hydrogen in -NH_2_ might lead to the formation of P_2_. Also, break of the S–N bond (sulfonamide bond) of P_1_/P_2_ could produce P_3_. Then, the ring-opening product (P_4_) could be produced via the cleavage of N–O bond in oxazol ring (P_3_)^36,78^.

### Comparison of SMZ degradation PFC System with some reports

Table. 1 presents some previous reports for comparison about the PFC system. This work employed Cu_2_O/CuO/Cu mesh photocathode in an easy one-step synthesis, which is cheaper and more accessible compared to many studies that use platinum as cathode. Meanwhile, the use of nickel foam as a support layer with high electrical conductivity and due to its high surface area increases electron transfer and enhances the photocatalytic activity of UiO-66-NH_2_-TiO_2_/NiF as an attractive photoanode with a synthesis of an easy step is used. The commercialization of the PFC system is an important question in this field, which the results of this article can partially answer.

**Table 1:** Some previous reports for comparison about the PFC system.

Anode	Cathode	Light source	Pollutant	V_ocp_ (v)	Power density µw/cm^2^	Degradation efficiency (%)	References
ZnO/C	P_t_/C	UVA	Reactive Green 19	~ 0.27	2.7	100% (480 min)	^79^
TiO_2_/ZnO/Zn	TiO_2_/CuO/Cu	UV-C lamp	Palm oil mill effluent	1.17	73.4	90% (120 min)	^80^
NiFe-LDH/BiVO_4_	Cu_2_O/Cu	300W Xe	Methyl blue	0.647	73	81% (360 min)	^81^
AgI-BiOI NFs	P_t_	Visible light	Formic acid	0.724	16.3	52.1% (240 min)	^82^
ZnO/Zn	CuO/Cu	UV	Methyl green	–	0.8	–	^83^
core/shell ZnFe_2_O_4_@ZnO	Air cathode	Solar light	COD	–	11.8	82.4% (5 h)	^84^
Er-Al co-doped Zn	Carbon cloth	Visible light	COD	0.55	1.83	69.7% (5 h)	^85^
CdS-TiO_2_/FTO	Commercial P_t_/C	Solar light	–	0.330	7	50% (12 h)	^86^
BiVO_4_/WO_3_	P_t_/C	Visible-light	–	0.150	8.58	87.2% (8 h)	^87^
BiVO_4_/UiO-66/TiO_2_/Ti	Cu_2_O/Cu	Visible light	Rhodamine B	0.498	2.9	91.5% (120 min)	^88^
UiO-66-NH_2_-TiO_2_/NiF	Cu/Cu_2_O	300W Xe	SMZ	0.644	16.98	91.7 (120 min)	This study
TiO_2_/NiF	Cu/Cu_2_O	300W Xe	SMZ	0.544	6.48	56% (120)	This study

## Conclusion

In this study, we used UiO-66-NH_2_-TiO_2_ as a photocatalyst for enhancing photocatalytic activity in the visible light region through one-step facile synthesis. The photocatalyst was deposited on a nickel foam substrate, and Cu_2_O/CuO/Cu mesh was utilized as the photocathode. Using nickel foam as a high-conductivity backing layer, and considering its large surface area, enhances electron transfer and strengthens the photocatalytic activity of UiO-66-NH_2_-TiO_2_/NiF. Optimization using a one-factor-at-a-time approach was performed to find the maximum photocatalytic degradation efficiency. Optimization using a one-factor method was performed to find the maximum degradation efficiency of light. The process factors have been optimized within the operational range (SMZ concentration = 20 ppm, pH = 6, radiation time = 120). The best performance of the PFC system was achieved under xenon light irradiation for the UiO-66-NH_2_-TiO_2_/NiF and TiO_2_/NiF composites, with efficiencies of 90.4% and 56%, respectively. This can be attributed to shorter band gaps, reduced recombination, and enhanced electron transfer from the anode to the cathode, which can increase the production of superoxide radicals at the cathode. The TOC analysis was 81.4% under optimal conditions for mineralization. Polarization curves (J-V) and power density (J-P) are measured to further investigate the photoanode in the PFC system, which showed that the maximum power in UiO-66-NH_2_-TiO_2_/NiF photoanode compared to TiO_2_/Ni is three times higher, indicating easier electron separation and transfer. The reusability of the UiO-66-NH_2_-TiO_2_/NiF anode with repeatable efficiency for removal and electrochemistry decreased by approximately 3.7% after five cycles (each cycle lasting 120 min).

## Data Availability

The datasets generated and analyzed during the current study were available from the corresponding author on reasonable request.
